# Automated segment-level coronary artery calcium scoring on non-contrast CT: a multi-task deep-learning approach

**DOI:** 10.1186/s13244-024-01827-0

**Published:** 2024-10-16

**Authors:** Bernhard Föllmer, Sotirios Tsogias, Federico Biavati, Kenrick Schulze, Maria Bosserdt, Lars Gerrit Hövermann, Sebastian Stober, Wojciech Samek, Klaus F. Kofoed, Pál Maurovich-Horvat, Patrick Donnelly, Theodora Benedek, Michelle C. Williams, Marc Dewey

**Affiliations:** 1 Department of Radiology, Charité—Universitätsmedizin Berlin, corporate member of Freie Universität Berlin, Humboldt-Universität zu Berlin, Berlin, Germany; 2 Artificial Intelligence Lab, Otto-von-Guericke-Universität, Magdeburg, Germany; 3 Department of Artificial Intelligence, Fraunhofer Heinrich Hertz Institute, Berlin, Germany; 4 Department of Electrical Engineering and Computer Science, Technical University of Berlin, Berlin, Germany; 5 BIFOLD—Berlin Institute for the Foundations of Learning and Data, Berlin, Germany; 6 Department of Cardiology and Radiology, Copenhagen University Hospital–Rigshospitalet, Copenhagen, Denmark; 7 Department of Clinical Medicine, Faculty of Health and Medical Sciences, University of Copenhagen, Copenhagen, Denmark; 8 Heart and Vascular Center, Semmelweis University, Budapest, Hungary; 9 Department of Radiology, Medical Imaging Center, Semmelweis University, Budapest, Hungary; 10 Department of Cardiology, Southeastern Health and Social Care Trust, Belfast, UK; 11 Department of Internal Medicine, Clinic of Cardiology, George Emil Palade University of Medicine, Pharmacy, Science and Technology, Targu Mures, Romania; 12 Centre for Cardiovascular Science, University of Edinburgh, Edinburgh, UK; 13 BIH (Berlin Institute of Health), DHZC (German Heart Center of the Charité) and DZHK (German Center for Cardiovascular Research), Berlin, Germany

**Keywords:** Coronary artery calcium scoring, Deep learning, Coronary CT, Multi-task learning, Active learning

## Abstract

**Objectives:**

To develop and evaluate a multi-task deep-learning (DL) model for automated segment-level coronary artery calcium (CAC) scoring on non-contrast computed tomography (CT) for precise localization and quantification of calcifications in the coronary artery tree.

**Methods:**

This study included 1514 patients (mean age, 60.0 ± 10.2 years; 56.0% female) with stable chest pain from 26 centers participating in the multicenter DISCHARGE trial (NCT02400229). The patients were randomly assigned to a training/validation set (1059) and a test set (455). We developed a multi-task neural network for performing the segmentation of calcifications on the segment level as the main task and the segmentation of coronary artery segment regions with weak annotations as an auxiliary task. Model performance was evaluated using (micro-average) sensitivity, specificity, F1-score, and weighted Cohen’s *κ* for segment-level agreement based on the Agatston score and performing interobserver variability analysis.

**Results:**

In the test set of 455 patients with 1797 calcifications, the model assigned 73.2% (1316/1797) to the correct coronary artery segment. The model achieved a micro-average sensitivity of 0.732 (95% CI: 0.710–0.754), a micro-average specificity of 0.978 (95% CI: 0.976–0.980), and a micro-average F1-score of 0.717 (95% CI: 0.695–0.739). The segment-level agreement was good with a weighted Cohen’s *κ* of 0.808 (95% CI: 0.790–0.824), which was only slightly lower than the agreement between the first and second observer (0.809 (95% CI: 0.798–0.845)).

**Conclusion:**

Automated segment-level CAC scoring using a multi-task neural network approach showed good agreement on the segment level, indicating that DL has the potential for automated coronary artery calcification classification.

**Critical relevance statement:**

Multi-task deep learning can perform automated coronary calcium scoring on the segment level with good agreement and may contribute to the development of new and improved calcium scoring methods.

**Key Points:**

- Segment-level coronary artery calcium scoring is a tedious and error-prone task.
- The proposed multi-task model achieved good agreement with a human observer on the segment level.
- Deep learning can contribute to the automation of segment-level coronary artery calcium scoring.

**Graphical Abstract:**

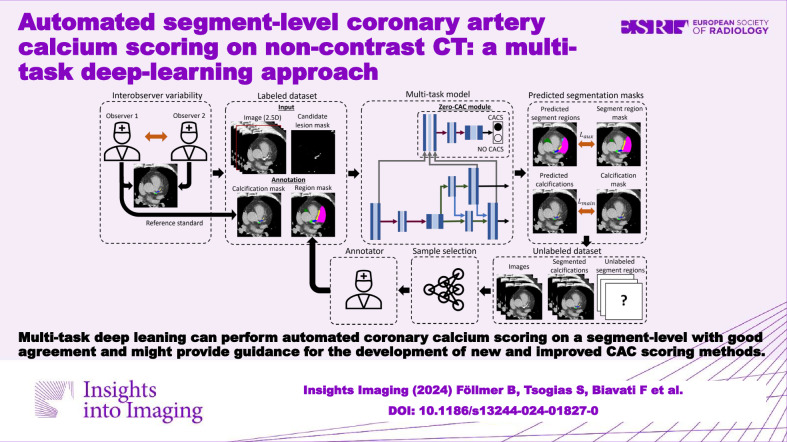

## Introduction

Coronary artery calcium (CAC) is an important feature of coronary atherosclerosis [1] and can be assessed using widely available non-contrast computed tomography (CT). The Agatston CAC score [2] is the most commonly used measure of CAC and has a high predictive value for subsequent cardiovascular events [3]. Clinical CAC scores are traditionally reported on the patient level and occasionally on the vessel level but only very rarely on the segment level, which is time-consuming because it involves additional manual efforts to separate calcifications and assign them to coronary segments [4]. Traditional CAC scoring methods are limited by the fact that they do not account for features such as the regional distribution of CAC and the number of calcified lesions or affected coronary artery segments [5, 6]. Several studies suggest that it would be worthwhile to investigate associations between the distribution of calcifications in the coronary artery tree and cardiovascular disease [7, 8]. A subanalysis of the Multi-Ethnic Study of Atherosclerosis (MESA) has shown that diffuse multivessel subclinical atherosclerosis is associated with coronary heart disease and cardiovascular disease [9]. Other studies showed that left main CAC was associated with increased mortality risk [8, 10]. Williams et al [11] have shown that automated assessment of vessel-specific coronary calcification quantification provides important prognostic information, raising the question of whether a segment-based calcium scoring methodology could further improve prognostic value [5]. However, manual CAC scoring on the segment level is a much more time-consuming task than traditional CAC scoring on the vessel level. The high costs of annotation and susceptibility to errors make it necessary to use deep-learning (DL)-based automated segment-level calcium scoring methods for large-scale data analysis. A fully automated approach to CAC scoring [11, 12] on the coronary segment level would facilitate the exploration of more sophisticated and granular calcium scoring methods for clinical decision-making. Such methods would also provide precise information on the location and distribution of calcifications, thereby contributing to a more accurate prediction of the risk of subsequent cardiovascular events.

Föllmer et al [13] have shown that learning the segmentation of coronary calcifications and segmentation of coronary artery regions in parallel on the vessel level improves CAC scoring performance and achieves high agreement between a DL network and the human reader reference standard. However, methods must be able to effectively handle image noise and accurately identify patients with zero CAC [14].

In this work, we propose a DL model that (1) performs calcium scoring on the segment level (13-segment model adapted from the Society of Cardiovascular Computed Tomography (SCCT) segmentation diagram [6]) as main task, (2) performs segmentation of coronary segment regions as auxiliary learning task [15], and (3) extends the multi-task model by a subsequential zero-CAC module to effectively distinguish between image slices with and without CAC, even in noisy conditions.

## Methods

### Study population

**Fig. 1:**
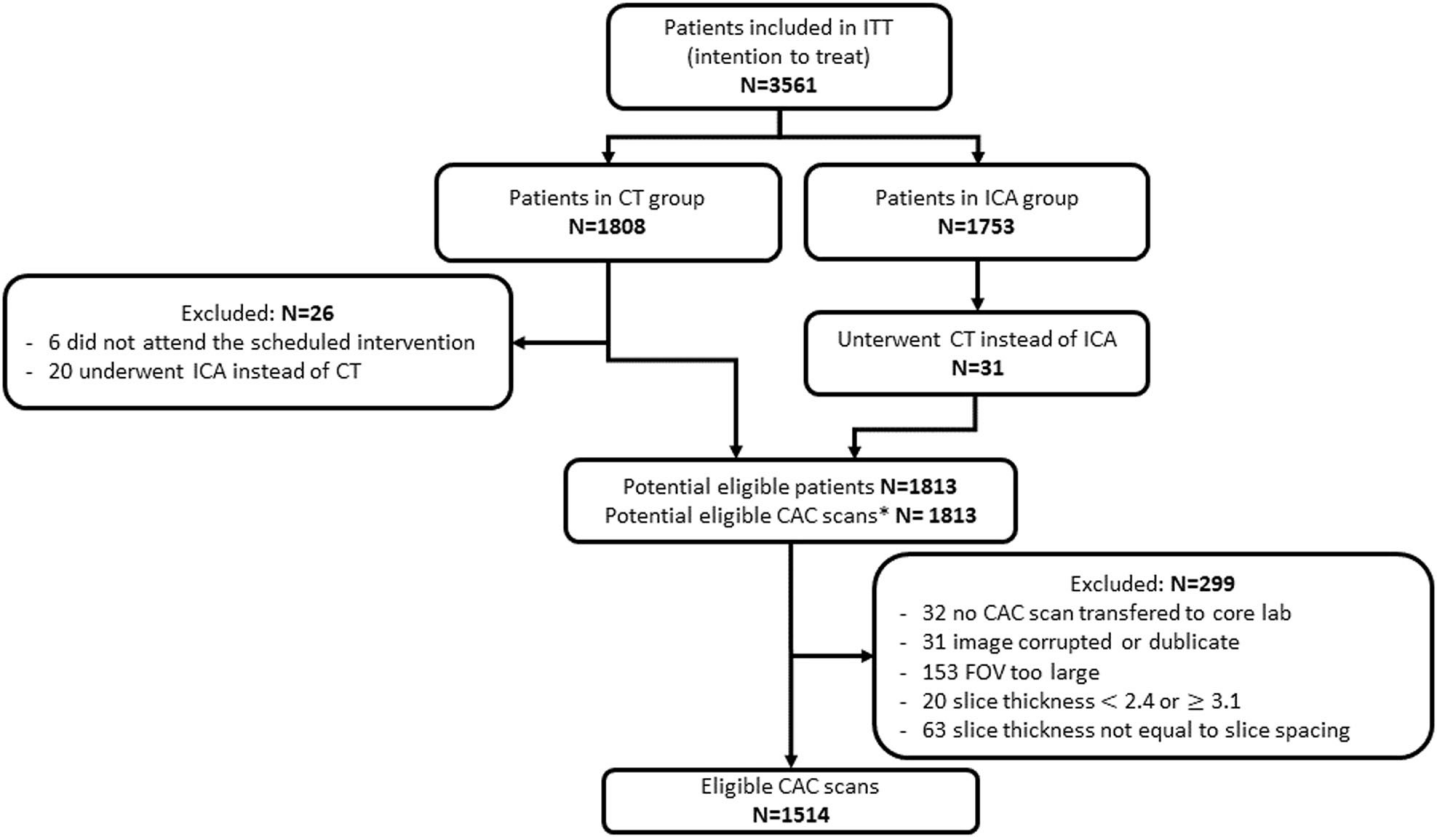
Flowchart of dataset selection from the DISCHARGE dataset

The dataset consists of 1514 ECG-gated, non-contrast CT scans performed on patients enrolled in the multicenter randomized controlled DISCHARGE trial [16–18] (NCT02400229). Examinations were performed at 26 clinical sites across Europe using CT scanners with at least 64-slice detectors from four different vendors. An Agatston score was determined using non-contrast cardiac CT scans with a slice thickness of 3.0 mm. We included all non-contrast CT scans with slice thicknesses ranging from 2.4 to 3.0 mm and excluded CT scans with smaller slice thicknesses because they might affect calcium scoring reproducibility due to increased noise. It is important to note that calcium scans with metal artifacts or anatomical abnormalities were not excluded from the dataset. A flowchart of the data selection process is presented in Fig. 1. Baseline characteristics of the selected patients are compiled in Table 1.

**Table 1:** Baseline characteristics of the training/validation set and test set. Continuous variables are presented as mean ± standard deviation

	Training/validation set (*n* = 1059)	Test set (*n* = 455)	*p*-value
*Patient characteristics*			
Women	55.5% (588/1059)	56.0% (255/455)	0.39
Age (years)	59.8 ± 10.1	60.4 ± 10.6	0.59
Body mass index (kg/m^2^)	28.8 ± 5.1	29.2 ± 5.4	0.16
*Scanner*			
GE medical systems	158 (14.9%)	80 (17.6%)	-
Philips	126 (11.9%)	61 (13.4%)	-
Siemens	418 (39.5%)	163 (35.8%)	-
Canon	357 (33.7%)	151 (33.2%)	-
*Risk factors*			
Hypertension	60.2 (637/1059)	60.9 (277/455)	0.26
Diabetes	14.4 (152/1059)	12.1 (55/455)	0.25
Hyperlipidemia	50.1 (531/1059)	46.8 (213/455)	0.06

All values are expressed as the mean ± standard deviation or number (%)

### Reference standard

The dataset containing 1514 calcium scoring CTs was randomly divided into three parts: 60% (908/1514) for training, 10% (151/1514) for validation, and 30% (455/1514) for testing.

Coronary segmentation diagrams [19], such as the SCCT coronary segmentation diagram [6], were defined based on the visibility of coronary artery segments in contrast-enhanced CT angiography (CCTA). Defining a segmentation diagram for non-contrast calcium scoring CT is challenging due to the lack of contrast between coronary arteries and surrounding tissue. Differentiation of side branches such as segment 12 (first obtuse marginal) and segment 14 (second obtuse marginal) or segments 16a (posterior descending artery from left circumflex artery, PDA-LCx) and 16b (posterior-lateral branch from left circumflex artery, PLB-LCx) is very challenging on non-contrast CT. Consequently, we modified the SCCT coronary segmentation diagram [6] for CAC scoring on the segment level as follows.

**Fig. 2:**
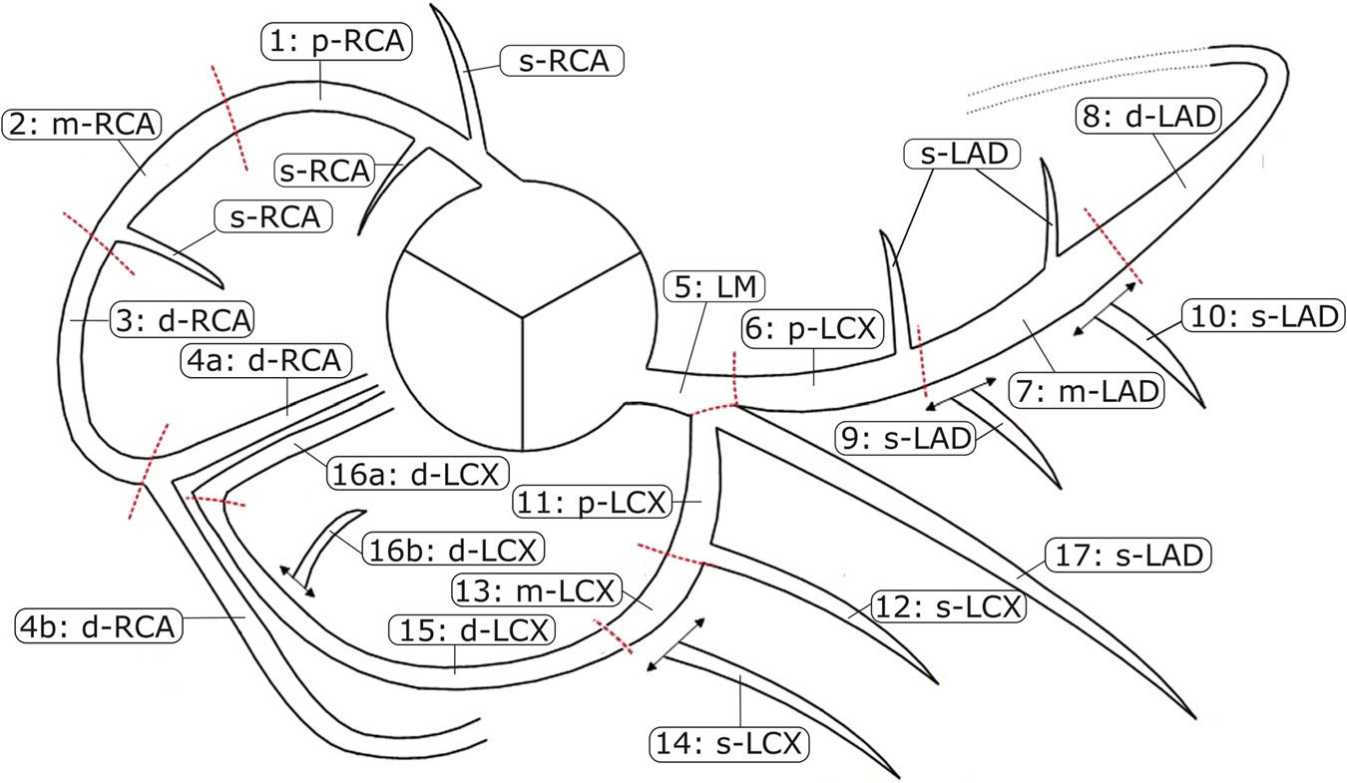
Adapted SCCT coronary segmentation diagram for segment-level calcium scoring. All side branches of the RCA are summarized as s-RCA, while segments 3, 4a, and 4b are labeled as d-RCA. Segments 9, 10, and 17 are combined into s-LAD. Segments 12 and 14 are combined into s-LCX, and segments 15, 16a, and 16b are combined to segment d-LCX

Coronary vessels were divided into proximal, mid, distal, and side branches. The adapted SCCT coronary segmentation diagram consists of 13 segments, including the left main (LM) and proximal, mid, distal, and side branches of the right coronary artery (RCA), left anterior descending (LAD), and left circumflex (LCX). The ramus intermedius, diagonal 1, and diagonal 2 are defined as LAD side branches (s-LAD). The first obtuse marginal (OM1) and second obtuse marginal (OM2) are defined as LCX side branches (s-LCX). Segments PLB-LCx and PDA-LCx are defined as LCX distal segments (d-LCX). The posterior-lateral branch of the right coronary artery (PLB-RCA) and the posterior descending artery from the right coronary artery (PDA-RCA) is defined as the RCA distal segment (d-RCA). The adapted SCCT coronary segmentation diagram is shown in Fig. 2.

The reference standard for the training, validation and test set of the model for the segmentation of CAC on the segment level (main task) was provided by a trained reader with three years of experience.

To evaluate the performance of the model in comparison with a human observer, we performed an interobserver variability analysis between the first observer (reference standard) and a second observer. The second observer was a trained reader with one year of experience who performed further segment-level CAC segmentation of the test set on the segment level.

**Fig. 3:**
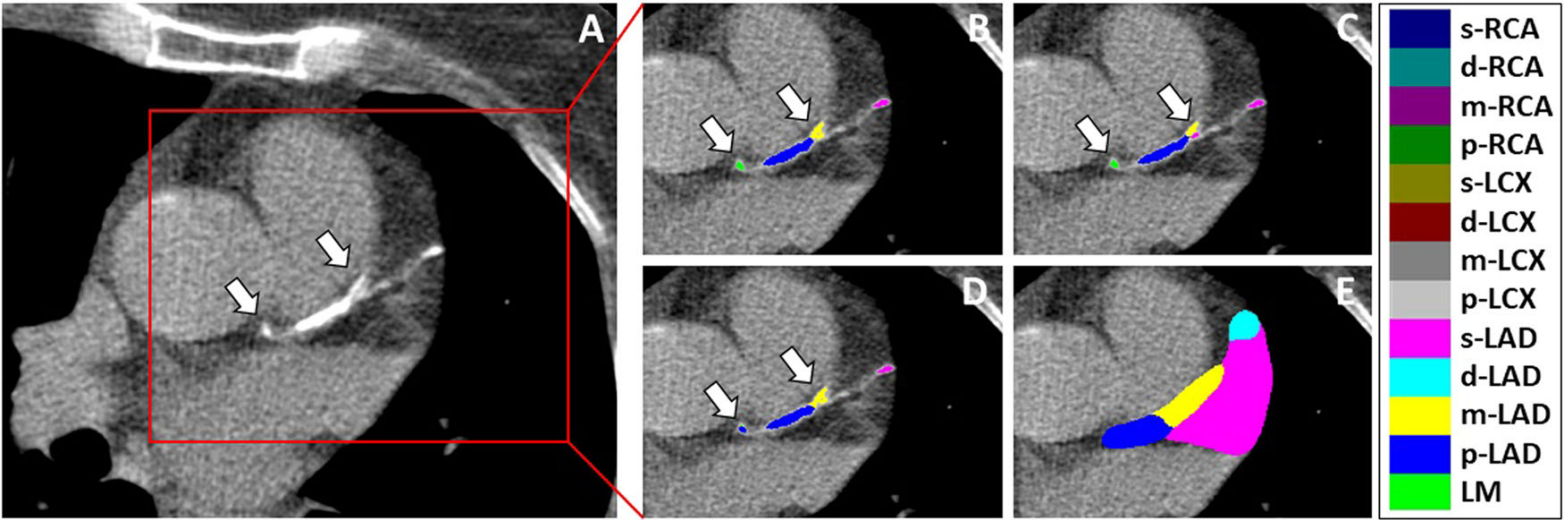
75-year-old female patient with coronary calcifications. **A** Image slice with coronary calcifications in the LM, p-LAD, m-LAD, d-LAD, and s-LAD. **B** Annotations for segment-level calcium scoring provided by the first observer. **C** Annotations for segment-level calcium scoring provided by the second observer. **D** Model prediction of segment-level calcium scores. **E** Model prediction of segment regions. Calcifications that were inconsistently assigned to the p-LAD and m-LAD by the two observers and small, misclassified calcifications that were incorrectly assigned to the p-LAD by the model are highlighted by arrows

Our goal was to ultimately develop a multi-task model for automated segmentation of coronary calcifications and coronary artery regions. To learn coronary artery regions not only from CTs with CAC but also from CT scans without CAC, we performed weak annotations of coronary segment regions (provided by a trained imaging scientist) using 3D Slicer [20] and made the algorithm learn this information as an auxiliary task. An example of an axial CT image that was annotated and the segmentations predicted by the model are presented in Fig. 3. Details of the annotation process are provided in Supplementary Material B.

### Model development and active learning training procedure

**Fig. 4:**
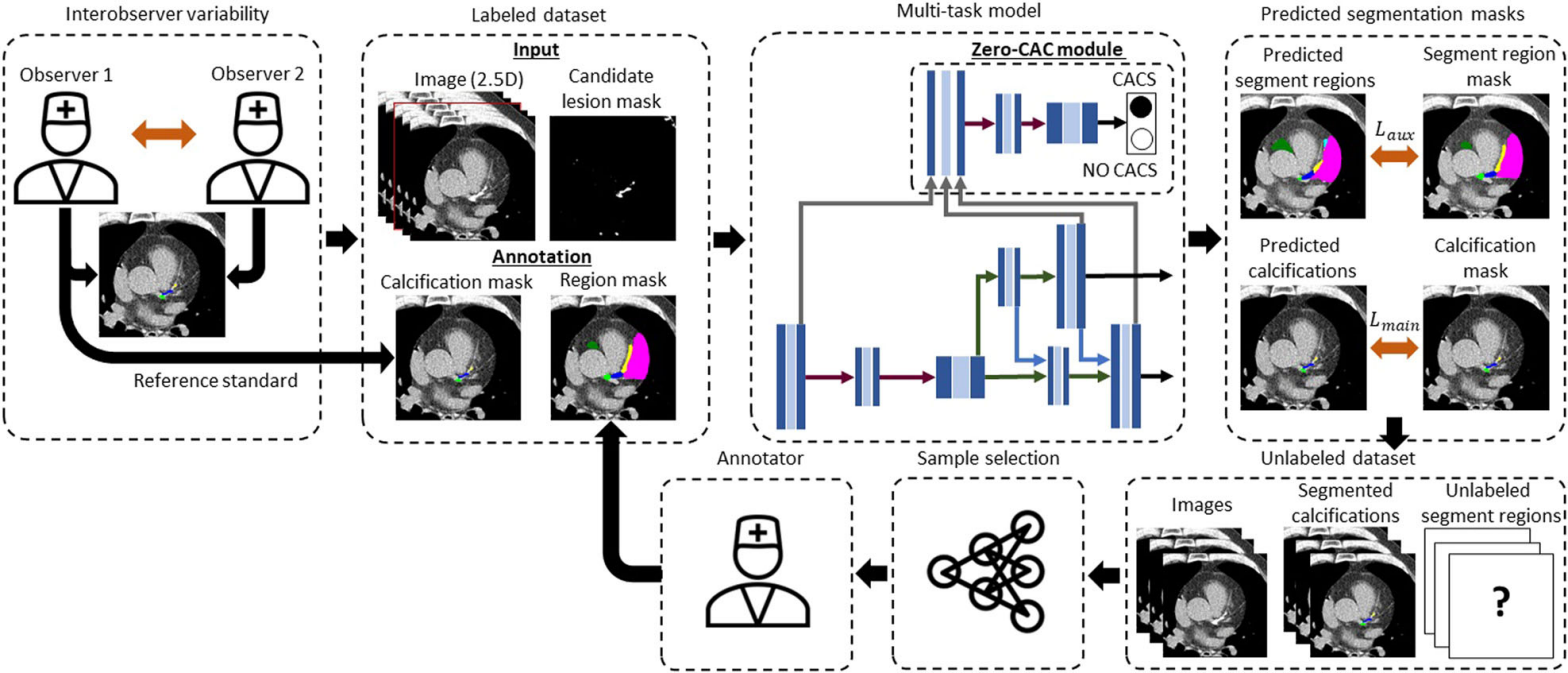
Diagram of the multi-task deep-learning procedure for segmentation of coronary calcium on the segment level and weak segmentation of coronary artery segment regions. The model was trained using the normalized image slices (2.5D) and the candidate lesion mask as input. The model selected the most informative samples from the unlabeled segment region set in multiple sampling rounds. The annotator corrected pseudo-labels of predicted segment regions, which served as training samples for the next training round. After completion of the active learning procedure, the zero-CAC module was trained to distinguish slices with and without CAC in order to improve the identification of zero-CAC patients

A multi-task DL model was developed that would perform segmentation of coronary calcifications on the segment level (main task) and segmentation of segment regions (auxiliary task) at the same time inspired by Föllmer et al [13]. The input tensor of the network was created by concatenating five consecutive slices, which were normalized via min-max normalization between − 2000 and 1300 HU (2.5D approach) [21]. Additionally, a candidate lesion mask was appended, which was created by thresholding the CT images with a constant threshold of 130 HU [2]. In the initial training round, weak segment region annotations were provided for only 100 randomly selected image slices. We used the uncertainty-weighted loss method proposed by Cipolla et al [22] to combine the loss of the main task, ${L}_{{main}}$, and the loss of the auxiliary task, ${L}_{{aux}}$, for multi-task model training (Fig. 4). To address the issue of annotation imbalance, we trained the model with a batch of 12 slices with and without calcifications, as well as with and without region annotations. Each batch comprises 12.5% of slices with both calcifications and region annotations, 12.5% of slices with calcifications and without region annotations, 37.5% of slices without calcifications but with region annotations, and 37.5% of slices without calcifications and region annotations. For samples without region annotations, we set the loss for region segmentations to zero.

To build the annotated dataset for weak region annotations, we used active learning [23–27] and increased the size of the labeled dataset in ten active learning rounds by labeling only most informative samples [28]. The most informative CT slices for weak annotation were selected using a sampling method that combines uncertainty-based sampling [25] and Fisher information correlation [26] between the main task and the auxiliary task. During each sampling round, a subset of the most uncertain samples identified for the main task ${{{{\boldsymbol{X}}}}}_{{{{\boldsymbol{M}}}}} {{\subseteq }}{{{{\boldsymbol{X}}}}}_{{{{\boldsymbol{U}}}}}$ and the most uncertain samples identified for the auxiliary task ${{{{\boldsymbol{X}}}}}_{{{{\boldsymbol{A}}}}}{{\subseteq }}{{{{\boldsymbol{X}}}}}_{{{{\boldsymbol{U}}}}}$ were selected from the unlabeled dataset using Monte Carlo dropout for uncertainty estimation [25]. Based on the Fisher maximum information correlation, samples $\widetilde{x}$ are iteratively selected $\widetilde{x}={{{{\rm{argmax}}}}}_{{x\in {{{\boldsymbol{X}}}}}_{{{{\boldsymbol{A}}}}}}{\rho }_{F}\left({{{{\boldsymbol{X}}}}}_{{{{\boldsymbol{M}}}}},\,{{{{\boldsymbol{X}}}}}_{{{{\boldsymbol{B}}}}}\cap x\right)$ for the batch ${{{{\boldsymbol{X}}}}}_{{{{\boldsymbol{B}}}}}$. The Fisher information correlation is defined as

$${\rho }_{F}\left({{{{\boldsymbol{X}}}}}_{{{{\boldsymbol{M}}}}},\,{{{{\boldsymbol{X}}}}}_{{{{\boldsymbol{B}}}}}\cap \widetilde{x}\right)=\frac{{i}_{C}{\left({f}_{M,A}\right)}_{{\theta }_{k}}}{\sqrt{{i}_{F}{\left({f}_{M}\right)}_{{\theta }_{k}}{i}_{F}{\left({f}_{A}\right)}_{{\theta }_{k}}}}$$

with ${i}_{F}{\left({f}_{M}\right)}_{{\theta }_{k}}$ and ${i}_{F}{\left({f}_{A}\right)}_{{\theta }_{k}}$ being the Fisher information of the samples ${{{{\boldsymbol{X}}}}}_{{{{\boldsymbol{M}}}}}$ and ${{{{\boldsymbol{X}}}}}_{{{{\boldsymbol{A}}}}}$, respectively. ${i}_{C}{({f}_{M,A})}_{{\theta }_{k}}$ is the average scalar product between the Fisher scores of the samples ${{{{\boldsymbol{X}}}}}_{{{{\boldsymbol{M}}}}}$ and ${{{{\boldsymbol{X}}}}}_{{{{\boldsymbol{A}}}}}$. The sample selection strategy is described in detail in Supplementary Material A. After each sampling round, the model was fine-tuned using the labeled dataset from the preceding round. After completion of the active learning procedure, we retrained the model from scratch to ensure generalizability and avoid overfitting by model warm start [29]. Compared with the earlier version proposed by Föllmer et al [13], we extended the network by the addition of a subsequential zero-CAC patient [14, 30] classification network, which explicitly learned to distinguish between slices with CAC and without any CAC. The model was trained using the image slices and the predictions of the multi-task model to learn the identification of zero-CAC patients from noisy images. Additionally, ablation experiments were conducted on the zero-CAC module, with the results presented in Supplementary Table S5. The model diagram and a detailed overview of model architecture are presented in Fig. 4 and Supplementary Fig. S1, respectively. The model hyperparameters we used are compiled in Supplementary Table S1. Training of this model was performed using the masks of calcifications on the segment level from the entire training set and weak annotations from the small, annotated dataset of calcifications and segment regions.

## Results

### Performance of the multi-task model

The model’s performance was assessed using the DISCHARGE test set of 455 patients (mean age 60.4 ± 10.6 years, 56.0% female). The baseline characteristics of the patients are presented in Table 1.

For the DISCHARGE test set, the model assigned 73.2% (1316/1797) of all correctly identified calcifications to the correct coronary segment class. To compare classification agreement, we employed a weighted Cohen’s *κ*, which considers uncertainties in segment boundaries by assigning a weight of 0.5 to the misclassification of adjoining segments and a weight of 1.0 to all other misclassifications. The weighting matrix is shown in Supplementary Table S2. The model achieved a weighted Cohen’s *κ* of 0.808 (95% CI: 0.790–0.824) [31] on the segment level compared to the reference standard (first observer). The confusion matrix for segment classification is presented in Table 2, while the confusion matrix for vessel classification is provided in Supplementary Table S3. The average micro-F1-score for multiclass segmentation of calcified lesion volume was 0.732 (95% CI: 0.711–0.754) on the segment level and 0.942 (95% CI: 0.931–0.952) on the vessel level. Detailed results regarding model performance on the segment level for multiclass segmentation, including intraclass correlation coefficient (ICC), precision, sensitivity, specificity, and F1-score, are presented in Table 3. Performance results are provided for the calcified volume and the number of calcified lesions. Supplementary Table S4 compiles model performance on the vessel level.

**Table 2:** Confusion matrix of the test set for the assignment of calcifications (number of calcified lesions) to the correct coronary artery segment. The model achieved good agreement with a weighted Cohen’s *κ* of 0.808 (95% CI: 0.790–0.824) for assigning calcifications to the correct segment class

	Automated segment prediction													
Reference^a^	p-RCA	m-RCA	d-RCA	s-RCA	LM	p-LAD	m-LAD	d-LAD	s-LAD	p-LCX	m-LCX	d-LCX	s-LCX	Total
**p-RCA**	300	17	0	0	0	0	0	1	1	0	0	0	0	319
**m-RCA**	31	62	13	0	0	0	0	0	0	0	0	0	0	106
**d-RCA**	3	6	134	0	0	0	0	0	0	0	0	2	0	145
**s-RCA**	4	6	0	0	0	0	0	0	0	0	0	0	0	10
**LM**	0	0	0	0	79	24	0	0	1	6	0	0	0	110
**p-LAD**	0	0	0	0	26	204	49	0	6	1	0	0	0	286
**m-LAD**	0	0	0	0	0	74	123	18	15	0	0	0	0	230
**d-LAD**	0	0	0	0	0	5	48	59	5	0	0	0	1	118
**s-LAD**	0	0	0	0	4	14	11	5	102	4	0	0	1	141
**p-LCX**	1	0	1	0	20	0	0	0	5	177	8	0	9	221
**m-LCX**	0	0	0	0	0	0	0	0	0	4	17	0	7	28
**d-LCX**	0	0	6	0	0	0	0	0	0	0	0	14	0	20
**s-LCX**	0	0	1	0	0	0	0	0	0	10	7	0	45	63
**Total**	339	91	155	0	129	321	231	83	135	202	32	16	63	1797

^a^ Reference segment diagram definition: p-RCA includes proximal RCA; m-RCA includes mid RCA; d-RCA includes distal RCA, PDA-RCA, PLB-RCA, s-RCA includes side branches of the RCA; p-LAD includes proximal LAD; m-LAD includes mid LAD; d-LAD includes distal LAD; s-LAD includes side branches of the LAD, Ramus intermedius, Diagonal 1, Diagonal 2; p-LCX includes proximal LCx; m-LCX includes mid LCx, d-LCX includes distal LCx, PDA-LCx, and PLB-LCx; s-LCX includes side branches of LCx, Obtuse marginal 1 and Obtuse marginal 2

**Table 3:** Performance of the model on the DISCHARGE test set for multiclass segmentation on the segment level and binary segmentation of coronary calcifications. Performance results are provided as intraclass correlation coefficient (ICC), precision, sensitivity, specificity, and F1-score for CAC segmentation on the volume level (vol.) and lesion level (no.).

SCCT segment number	Adapted segment model	Number of calcified voxel/lesions	ICC (vol./num.)	Precision (vol./num.)	Sensitivity (vol./num.)	Specificity (vol./num.)	F1-score (vol./num.)
**1**	**p-RCA**	36,886/346	0.88/0.9	0.84/0.88	0.95/0.94	0.96/0.97	0.83/0.81
**2**	**m-RCA**	16,346/131	0.35/0.53	0.65/0.68	0.44/0.58	0.98/0.98	0.43/0.50
**3, 4a/4b**	**d-RCA**	16,620/182	0.69/0.78	0.85/0.86	0.90/0.92	0.99/0.99	0.73/0.73
**RCA-side**	**s-RCA**	603/15	-/-	-/-	0.00/0.00	1.00/1.00	0.00/0.00
**5**	**LM**	13,140/133	0.67/0.71	0.64/0.61	0.67/0.72	0.97/0.97	0.52/0.53
**6**	**p-LAD**	41,787/303	0.80/0.59	0.70/0.64	0.72/0.71	0.91/0.92	0.69/0.61
**7**	**m-LAD**	24,019/241	0.72/0.60	0.50/0.53	0.54/0.53	0.92/0.93	0.49/0.50
**8**	**d-LAD**	7903/134	0.48/0.58	0.66/0.71	0.40/0.50	0.99/0.99	0.43/0.52
**9, 10, 17**	**s-LAD**	9832/160	0.85/0.83	0.67/0.76	0.66/0.72	0.98/0.98	0.63/0.64
**11**	**p-LCX**	21,363/241	0.73/0.81	0.85/0.88	0.79/0.80	0.98/0.98	0.71/0.76
**13**	**m-LCX**	3002/29	0.83/0.57	0.67/0.53	0.62/0.61	1.00/0.99	0.54/0.50
**15, 16a/16b**	**d-LCX**	1355/29	0.47/0.68	0.90/0.88	0.40/0.70	1.00/1.00	0.49/0.60
**12, 14**	**s-LCX**	4742/89	0.67/0.73	0.57/0.71	0.70/0.71	0.99/0.99	0.53/0.54
**Binary CAC segmentation**	-	197,598/2033	0.91/0.97	0.93/0.86	0.88/0.88	1.00/1.00	0.90/0.87

		Number of calcified voxel/lesions	Micro ICC^a^ (vol./num.)	Micro Precision^a^ (vol./num.)	Micro sensitivity^a^ (vol./num.)	Micro specificity^a^ (vol./num.)	Micro F1-score^a^ (vol./num.)
**Micro segment score**	-		0.87/0.82	0.72/0.73	0.72/0.73	0.98/0.98	0.72/0.73

^a^ Micro metrics are calculated based on the classification of correctly detected calcification into the correct segment class (missed calcifications of the model are not included in the metric)

The mapping from the SCCT coronary segmentation diagram to the adapted coronary segment model is highlighted in bold

**Fig. 5:**
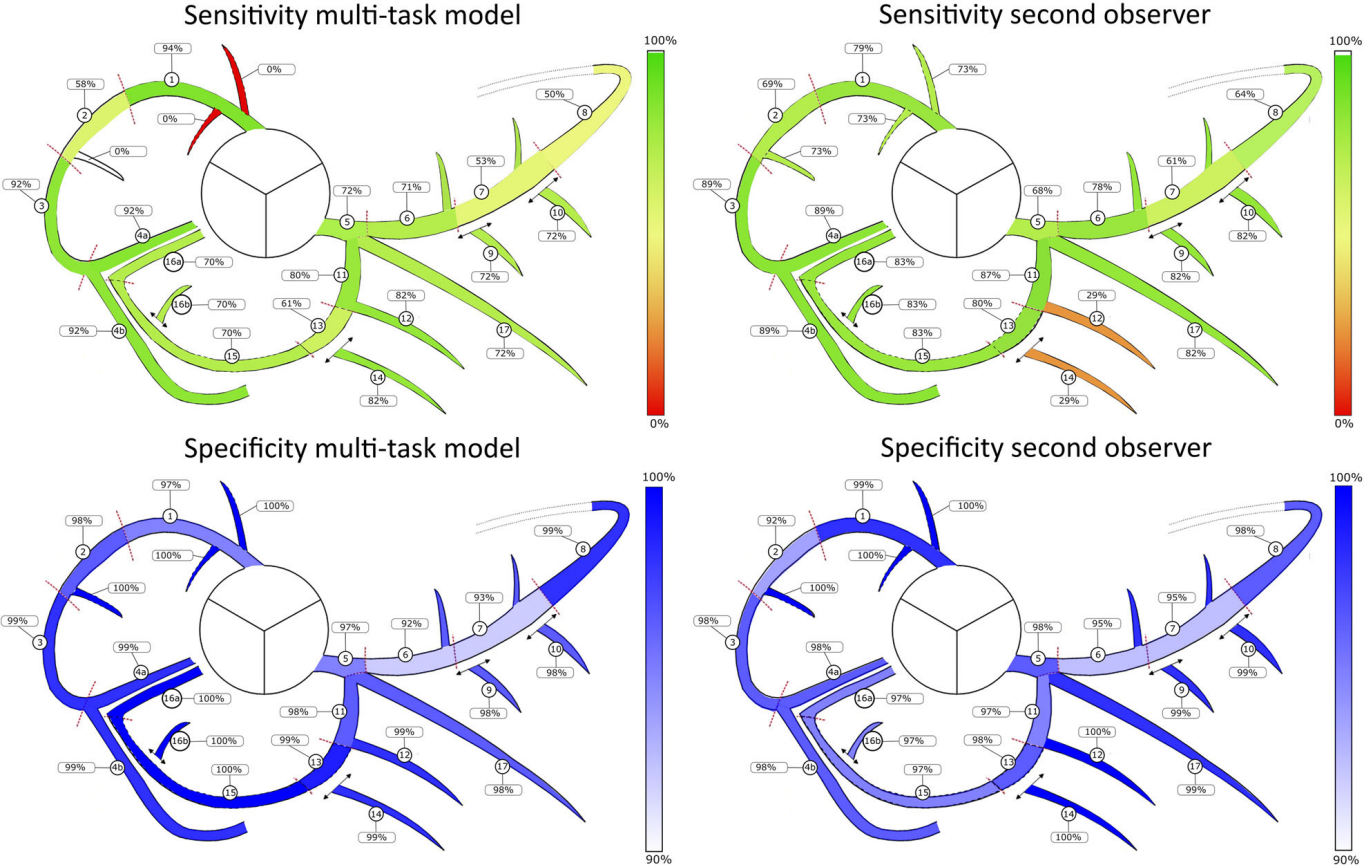
Comparison of the sensitivity and specificity of the multi-task model and the second observer for assignment of calcified lesions to the correct arterial segment using the adapted SCCT coronary segmentation diagram. Color intensity is a quadratic function of the performance with the range (0%, 100%) for sensitivity and (90%, 100% (for specificity)

On the segment level, the model achieved the highest sensitivities for the proximal RCA (0.94), distal RCA (0.92), and proximal LCX (0.80). Sensitivities were lowest for the side RCA (0.0), distal LAD (0.50), and mid LAD (0.53). Most misclassifications on the segment level occurred between proximal and mid segments (38.0% (183/481)) and between mid and distal segments (44.3% (213/481)). Sensitivities and specificities are provided in the coronary segmentation diagram in Fig. 5.

The F1-score for binary segmentation of calcified lesions was 0.90 for calcified volume and 0.87 for the number of calcified lesions. Risk categorization was based on the Agatston score with risk category I: 0 Agatston units (AU), group II: (1,100) AU, group III: (100,300), and group IV: > 300 AU. The model had an excellent agreement with the reference standard with a linear weighted Cohen’s *κ* of 0.909 (95% CI: 0.889–0.933). Most of the misclassifications were off by one category, mainly between risk category I (zero-CAC patient) and category II (1 ≤ CAC < 100). The confusion matrix for CVD risk categorization is provided in Table 4. To analyze the influence of the zero-CAC module presented in Fig. 4, we performed an ablation experiment, which revealed a linear weighted Cohen’s *κ* of only 0.868 (95% CI: 0.831–0.898) for CVD risk categorization using the model without the zero-CAC module. The confusion matrix for CVD risk categorization without the zero-CAC module is shown in Supplementary Table S5.

**Table 4:** Confusion matrix for agreement of CVD risk estimates for the DISCHARGE test set with a Cohen’s *κ* of 0.909 (95% CI: 0.880–0.933). Risk categorization was based on the Agatston score with risk categories I: 0, II: (1, 100), III: (100, 300), IV: > 300

Risk	I	II	III	IV	Total
I	162	30	2	0	194
II	2	113	5	1	121
III	0	1	62	3	66
IV	0	1	0	73	74
Total	164	145	69	77	455

### Interobserver variability for segment-level calcium scoring

Coronary calcium scoring on the segment level is a complex and time-consuming task, with a median annotation time of 1.85 min per scan (interquartile range, 0.65 to 3.05). Interobserver variability analysis revealed that the second observer outperformed the model, achieving an F1-score of 0.96 for calcification segmentation versus the model’s F1-score of 0.87. However, for the correct assignment of detected calcifications to their respective segment classes, the observer achieved a micro-F1-score of 0.76, which was only slightly higher than the model’s score of 0.72.

Model agreement with the first observer (reference standard) regarding this assignment task was good, with a weighted Cohen’s *κ* of 0.808 (95% CI: 0.790–0.824), which was only slightly lower than the agreement between the first and the second observer (0.809 (95% CI: 0.798–0.845)).

The detailed results are presented in Supplementary Table S6. Figure 5 compares model performance on the segment level with that of the second observer in terms of sensitivity and specificity. Differences in performance are primarily attributable to misclassifications between proximal and mid segments and between mid and distal segments. Note that the model was trained using the annotations of the first observer as the reference standard.

### Performance gain with respect to labeling time for auxiliary task annotation

We compared the performance of our multi-task model with that of a standard U-Net [32] for multiclass segment-level CAC scoring. The performance results of the U-Net are compiled in Supplementary Table S7. The U-Net achieved an F1-score for binary segmentation of only 0.83 on the volume level, which is already below state-of-the-art performance [12, 33], and a micro-average-F1-score of 0.581 (95% CI: 0.549–0.611) on lesion level which is much lower compared to our model with 0.73 (95% CI: 0.710–0.845). As the distribution of lesion classes was very imbalanced, the model did not learn the classification of calcified lesions in the m-RCA, s-RCA or m-LCX, d-LCX, or s-LCX and only assigned lesions to the majority classes.

## Discussion

In this study, we show that automated segment-level CAC scoring using DL achieves good results for the assignment of calcified lesions to the correct segment of the coronary artery tree. Specifically, the model presented here achieved good agreement with the first observer (reference standard), and agreement was only slightly below the agreement between the first and the second observer. The results demonstrate that our multi-task learning approach, which involves learning the auxiliary task of coronary artery region segmentation, effectively addresses the issue of learning from imbalanced datasets. Further research will be done to explore the potential applicability of the proposed method to similar medical image analysis problems. To reduce the burden of the time-consuming task of weak segment region annotation (approximately 2 min/scan), we used active learning to label only 2.3% of the image slices with segment regions. To address the issue of false positive noise segmentation as micro-calcification [12], which results in the false assignment of zero-CAC patients to higher risk categories, we expanded the network by adding a binary CAC classification module to differentiate slices with and without CAC. An ablation study demonstrated that the addition of the proposed zero-CAC module improved the agreement of patient risk categorization. The expanded model achieved an F1-score for binary segmentation of CAC of 0.907, which indicates a high performance compared to current state-of-the-art automated CAC scoring methods [12, 33, 34]. Both sensitivity and F1-scores were higher for proximal segments compared with side branches, indicating that the model may be particularly useful for automatically identifying proximal CAC, which is an independent marker of major adverse cardiovascular events [35]. The underrepresented segment class s-RCA was not learned by the model, and all 15 s-RCA lesions were misassigned to other RCA segment classes, calling for larger training sets and further model adaptation. Nevertheless, further interobserver variability analysis is necessary to assess the limitations of discriminating small side branches in non-contrast CT with and without the support of contrast-enhanced CTA, as this can be assumed as an upper bound on model performance when used as ground truth. Cardiovascular risk scores that quantify the total coronary atherosclerotic burden CCTA and include information on the localization of calcified lesions such as the CT-adapted Leaman score (CT-LeSc) [36, 37] have been shown to be an independent long-term predictor of cardiac events in CCTA. For non-contrast CT scans, the Eisner study has shown that the presence and extent of proximal CAC plaques provide strong independent prognostic information for predicting major adverse cardiovascular events (MACE) [35]. Blaha et al [10] have demonstrated that measures of calcified plaque distribution improve the ability of the traditional Agatston score in predicting coronary artery disease (CAD) events. Williams et al [11] have shown that vessel-specific automated DL-based CAC analysis can be performed accurately and provides important prognostic information. Biavati et al [18] have shown that in people with stable chest pain, zero-CAC patients had a very low risk of MACE. Higher CAC scores showed an increasing risk of obstructive CAD, revascularization, and MACE.

Coronary calcium scoring on the segment level might improve the predictive value of calcium scoring since information on calcium distribution across the segments of the coronary artery tree is not lost, and the presence of bifocal/trifocal calcified lesions can be considered in risk prediction [5]. The proposed model could also assist in CT angiography planning and serve as a gatekeeper to avoid unnecessary subsequent angiography in the presence of a high calcium score in high-risk segments. The high interobserver variability indicates that automation might also improve reproducibility in clinical practice.

For large-scale study of CAC scoring on the segment level, it is necessary to automate the time-consuming and error-prone annotation task. To the best of our knowledge, this is the first study investigating DL models for CAC scoring on the segment level.

Our study has several limitations. One limitation is the lack of consensus between the two observers. Thus, the annotations made by the first observer were used as the reference standard for model training. The high interobserver variability in scoring CAC on the segment level, particularly for side branches, could have had a negative impact since training with inaccurate labels has the potential to degrade model performance. Our analysis was conducted on images with a slice thickness exceeding 2.4 mm to align with the standard methodology proposed by Agatston et al [2]. However, we plan to modify our model to accommodate thin-slice reconstructions, with the objective of enhancing the discrimination of small distal branches. Future work will investigate whether model performance can be enhanced by using a training set annotated by multiple observers or an improved reference standard based on contrast-enhanced CT images to determine the exact location of calcified lesions in the coronary artery tree. We will also investigate the potential of our DL model to improve reproducibility and reduce interobserver variability in CAC scoring on a segment level by highlighting likely mislabeled lesions and visualizing model uncertainties.

## Conclusions

In this study, we propose a deep-learning model for coronary calcium scoring on a segment level. The model achieved good agreement with a human observer and has the potential to serve as a tool to further study the association between the distribution of calcifications in the coronary artery tree and cardiovascular risk in large imaging studies. The model presented here might provide guidance for the development of new and improved CAC scoring methods.

## Supplementary information

ELECTRONIC SUPPLEMENTARY MATERIAL

## Data Availability

The trained model and software code for model inference from this study can be found at https://github.com/Berni1557/SEGMENT-CACS/tree/main. The data is not publicly available due to privacy and ethical restrictions.

## Abbreviations

CAC: Coronary artery calcium
CAD: Coronary artery disease
CCTA: Coronary CT angiography
CI: Confidence interval
DL: Deep learning
HU: Hounsfield unit
ICC: Intraclass correlation coefficient
SCCT: Society of Cardiovascular Computed Tomography
