# Supplementary material for: Automated segment-level coronary artery calcium scoring on non-contrast CT: a multi-task deep-learning approach
Source: Insights Imaging. 2024 Oct 16;15:250. doi: 10.1186/s13244-024-01827-0 (PMC11484984; doi:10.1186/s13244-024-01827-0)
Supplement: Supplementary file 1 — ELECTRONIC SUPPLEMENTARY MATERIAL [file 13244_2024_1827_MOESM1_ESM.pdf]

Automated segment-level coronary artery calcium scoring on non-contrast CT: a multi-task deep learning approach

ELECTRONIC SUPPLEMENTARY MATERIAL

Supplementary Material A: Multi-task active learning selection strategy

For the learning task, we define segment-level calcium scoring as the main task and weak segmentation of the segment regions as the auxiliary task. Annotations for the segment-level CAC scoring task are provided for the whole dataset, and active learning is used to provide annotations for the auxiliary task. Unlabeled dataset $\boldsymbol{X_U} = \{(x, y_M\}$ consists of image slices (2.5D) x and annotations for the main task, y_M. Labeled dataset $\boldsymbol{X_S} = \{(x, y_M, y_A)\}$ consists of image slices with annotations for the main task, y_M , and annotations for the auxiliary task $, y_A$. In the active learning procedure, the multi-task model is initially trained with randomly selected annotated initial dataset $\boldsymbol{X_{S,init}} = \{(x, y_M, y_A)\}$ of 100 slices. During multiple sampling rounds, the most informative CT slices are selected using a sampling method that combines uncertainty-based sampling [1] and Fisher information correlation [2] between the main task and the auxiliary task. During each sampling round, a subset of the most uncertain samples identified for the main task $\boldsymbol{X_M} \subseteq \boldsymbol{X_U}$ and the most uncertain samples identified for the auxiliary task $\boldsymbol{X_A} \subseteq \boldsymbol{X_U}$ are selected from the unlabeled dataset. To estimate the uncertainty, we use Monte-Carlo dropout as performed by the standard STD mean sampling method [1]. A new informative batch of samples, $\boldsymbol{X_B}$ **,** is iteratively selected from $\boldsymbol{X_A}$ based on the Fisher information correlation coefficient $\rho_F(\boldsymbol{X_M}, \boldsymbol{X_B} \cap x)$ between dataset $\boldsymbol{X_M}$ and the batch of selected samples including slice candidate $\boldsymbol{X_B} \cap x$.

Fisher information $i_F(f_M)_{\theta_k}$of the uncertain main task samples and Fisher information $i_F(f_A)_{\theta_k}$ of the selected batch and the slice candidate are estimated using gradient embeddings from hard pseudo-labels [3] of model output f_M and f_A , respectively. The model architecture is shown in **Supplementary Figure S1**. The algorithm is presented in **Algorithm 1**.

$$i_F(f_M)_{\theta_k} = \frac{1}{\mathrm{N_M}} \sum_{x_M \in X_M} \frac{\partial \log f_M(x_M)}{\partial \theta_k} \frac{\partial \log f_M(x_M))}{\partial \theta_k} \tag{1}$$

$$i_F(f_A)_{\theta_k} = \frac{1}{|X_B| + 1} \sum_{x_B \in X_B \cap x} \frac{\partial \log f_A(x_B)}{\partial \theta_k} \frac{\partial \log f_A(x_B)}{\partial \theta_k} \tag{2}$$

$$i_C\left(f_{M,A}\right)_{\theta_k} = \frac{1}{|X_B| + 1} \sum_{x_B \in X_B \cap x} \frac{1}{\mathrm{N_M}} \sum_{x_M \in X_M} \frac{\partial \log f_A(x_B)}{\partial \theta_k} \frac{\partial \log f_M(x_M))}{\partial \theta_k} \tag{3}$$

$$\rho_F(\boldsymbol{X_M}, \boldsymbol{X_B} \cap \tilde{x}) = \frac{i_C\left(f_{M,A}\right)_{\theta_k}}{\sqrt{i_F(f_M)_{\theta_k} i_F(f_A)_{\theta_k}}} \tag{4}$$

Input: dataset with segment-level CAC annotation but without weak segment region annotation $\boldsymbol{X_U}$, number of sampling rounds K, number of selected most uncertain samples based on main task N_M, number of selected most uncertain samples based on auxiliary task N_A, number of informative batch samples N_B
Train the initial model on $\boldsymbol{X_{S,init}}$: $\boldsymbol{\theta}_0 \coloneqq \underset{\boldsymbol{\theta}}{\mathrm{argmin}}\ \mathrm{E}_{\boldsymbol{X_S}}[l(x, y_M, y_A)]$
for k=1, 2, …, K **do**
  Select the N_M most uncertain samples with respect to main task $\boldsymbol{X_M} \subseteq \boldsymbol{X_U}$,
  Select the N_A most uncertain samples with respect to auxiliary task $\boldsymbol{X_A} \subseteq \boldsymbol{X_U}$,
  $\boldsymbol{X}_B = \emptyset$
  for b=1, 2, 3, …, N_B **do**
    $\tilde{x} = \underset{x \in \boldsymbol{X_A}}{\mathrm{argmax}}\, \rho_F\ (\boldsymbol{X_M}, \boldsymbol{X_B} \cap x)$
    $\boldsymbol{X_B} \leftarrow \boldsymbol{X_B} \cap \tilde{x}$
  end for
  Query the annotator to obtain segmentations of weak segment region annotation for $\boldsymbol{X_B}$
  $\boldsymbol{X_S} \leftarrow \boldsymbol{X_S} \cap \boldsymbol{X_B}$
  Train the model on $\boldsymbol{X_S}$: $\boldsymbol{\theta_k} \coloneqq \underset{\boldsymbol{\theta}}{\mathrm{argmin}}\ \mathrm{E}_{\boldsymbol{X_S}}[l(x, y_M, y_A)]$
end for

Algorithm 1: Algorithm of the active learning procedure to select the most informative image slices to provide annotations for segment regions in multiple sampling rounds.

For all experiments, we selected a subset of $N_M = 1000$ and $N_A = 1000$ most uncertain samples, which corresponds to approximately 2.5% of the image samples in the training set. The model was trained in $K = 10$ active learning rounds, and we

stopped once performance seemed to plateau, as shown in **Supplementary Figure S2**. We chose an annotation budget of $N_B = 100$ slices, which is a realistic budget for practical active learning scenarios.

Supplementary Material B: Annotation process for coronary artery calcifications and coronary artery regions

The classes used for the coronary segment regions correspond to those for segment-level CAC scoring with an additional class for labeling of metal artifacts within the heart (e.g., stents, pacemakers). Since annotating segment regions is highly time-consuming, we used a more efficient active learning procedure. This annotation process labels segment regions for only a small subset of 2.3% (1000/44063) of the most informative image slices of the training set across 10 sampling rounds to improve the model's performance on the main task (segment-level calcium scoring). To annotate coronary calcifications on the segment level and coronary artery segment regions, we utilized an in-house developed semi-automatic segmentation module for 3D Slicer (version 5.2.2, https://www.slicer.org) [4]. Candidate lesions for annotation were identified by highlighting all voxels with attenuation above 130 Hounsfield units (HU). For model evaluation, calcified lesions were defined as connected voxels (6-connectivity) with a minimum volume of 1.5 mm^3. The two observers annotated all highlighted voxels of calcified lesions and assigned them to one of the 13 segment labels. Candidate lesions that were present in multiple neighboring segments were split, and voxels were annotated without overlap.

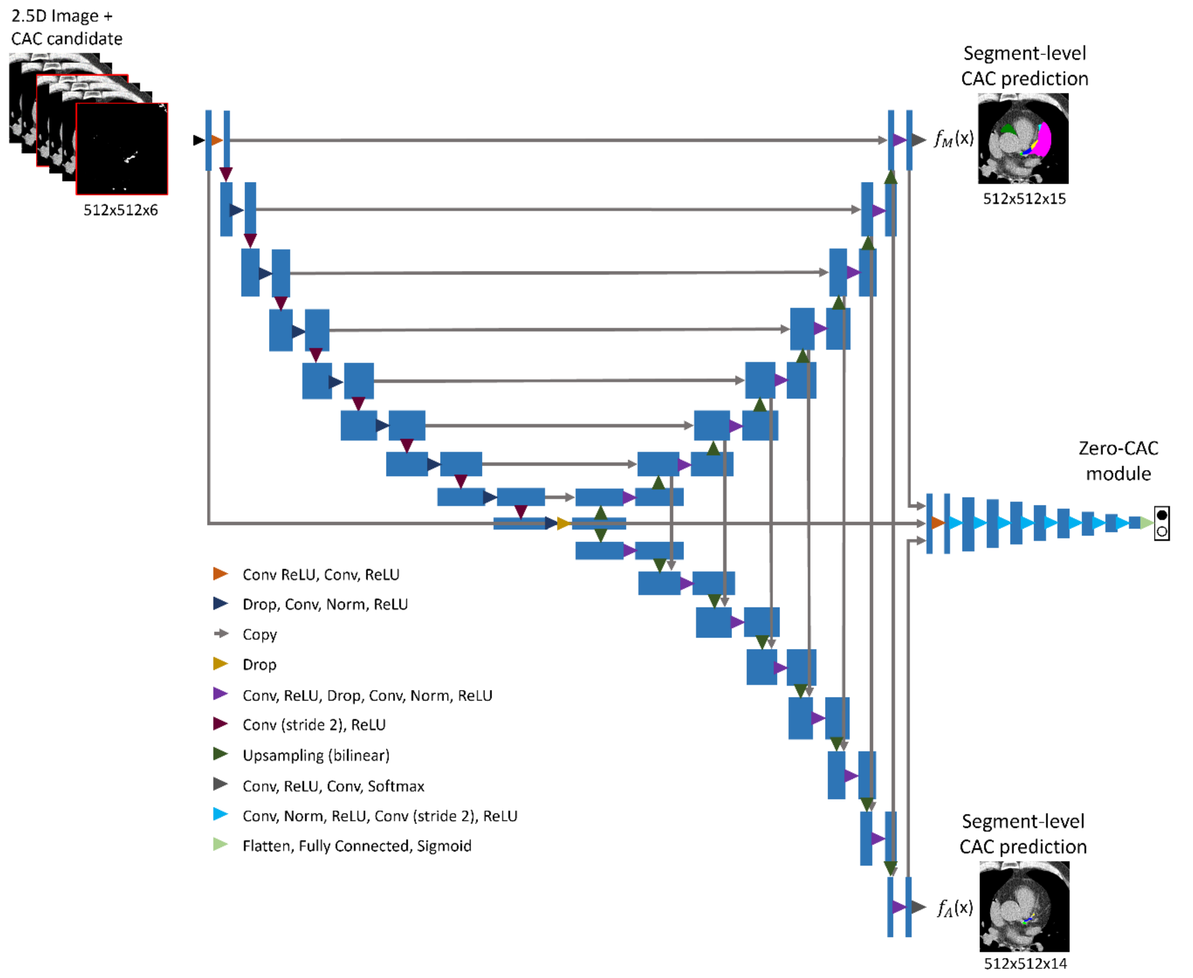

Supplementary Figure S1: Multi-task model for segmentation of coronary calcifications on the segment level (main task), segmentation of coronary artery segment regions (auxiliary task), and image slice-based binary CAC classification.

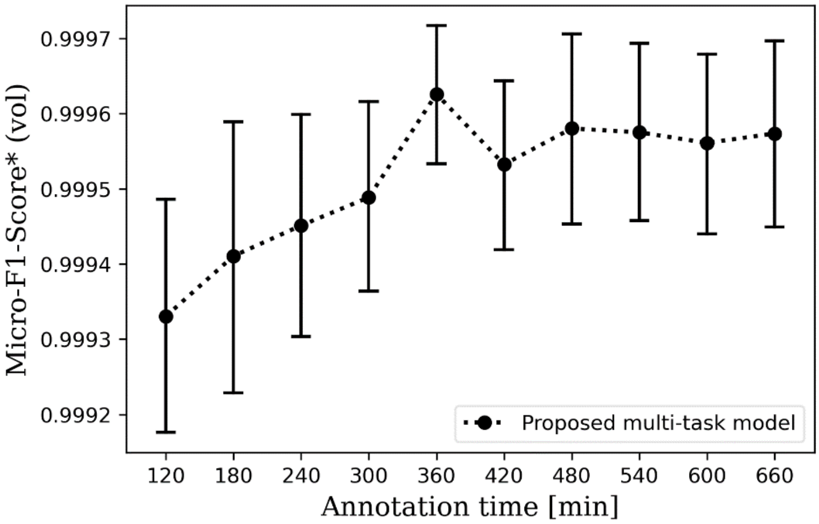

Supplementary Figure S2: Performance gain in terms of micro-F1-score (CAC volume) in 10 active learning rounds in terms of labeling time required for the auxiliary task (weak segment region annotation) for multiclass segment-level CAC scoring.
***The average micro-F1 score includes non-CAC voxels, as opposed to the average micro-F1 score in Table 4.**

Supplementary Table S1: Hyperparameters of the multi-task segmentation model and the zero-CAC module.

**Multi-task model hyperparameter**	
Optimizer	Adam optimizer
Learning rate (init)	0.001
Learning rate (fine-tuning)	0.0001
Learning rate decay rate	0.98
Regularization	L2norm
Dropout rate	0.5
Batch size	12
Loss	Uncertainty weighted loss with cross entropy [5]
Iterations (init)	41500
Iterations (fine-tune)	24900
**Zero-CAC module hyperparameter**	
Optimizer	Adam optimizer
Learning rate	0.001
Learning rate decay rate	0.98
Regularization	L2norm
Batch size	12
Loss	Cross entropy
Iterations	24900

Supplementary Table S2: Weighting matrix for estimation of the weighted Cohen's κ [6] on the segment level. Neighboring segments are weighted by 0.5, all other misclassifications are weighted by 1.0.

	**Multi-task DL model**												
**Reference**	**p-RCA**	**m-RCA**	**d-RCA**	**s-RCA**	**LM**	**p-LAD**	**m-LAD**	**d-LAD**	**s-LAD**	**p-LCX**	**m-LCX**	**d-LCX**	**s-LCX**
**p-RCA**	0.0	0.	1.	0.5	1.0	1.0	1.0	1.0	1.	1.0	1.0	1.0	1.0
**m-RCA**	0.5	0.	0.	0.5	1.0	1.0	1.0	1.0	1.	1.0	1.0	1.0	1.0
**d-RCA**	1.0	0.	0.	0.5	1.0	1.0	1.0	1.0	1.	1.0	1.0	1.0	1.0
**s-RCA**	0.5	0.	0.	0.0	1.0	1.0	1.0	1.0	1.	1.0	1.0	1.0	1.0
**LM**	1.0	1.	1.	1.0	0.0	0.5	1.0	1.0	1.	0.5	1.0	1.0	1.0
**p-LAD**	1.0	1.	1.	1.0	0.5	0.0	0.5	1.0	0.	0.5	1.0	1.0	1.0
**m-LAD**	1.0	1.	1.	1.0	1.0	0.5	0.0	0.5	0.	1.0	1.0	1.0	1.0
**d-LAD**	1.0	1.	1.	1.0	1.0	1.0	0.5	0.0	0.	1.0	1.0	1.0	1.0
**s-LAD**	1.0	1.	1.	1.0	1.0	0.5	0.5	0.5	0.	1.0	1.0	1.0	1.0
**p-LCX**	1.0	1.	1.	1.0	0.5	0.5	0.5	1.0	1.	0.0	0.5	1.0	0.5
**m-LCX**	1.0	1.	1.	1.0	1.0	1.0	1.0	1.0	1.	0.5	0.0	0.5	0.5
**d-LCX**	1.0	1.	1.	1.0	1.0	1.0	1.0	1.0	1.	1.0	1.0	0.0	0.5
**s-LCX**	1.0	1.	1.	1.0	1.0	1.0	1.0	1.0	1.	0.5	0.5	0.5	0.0

*** Reference segment diagram definition: p-RCA includes proximal RCA; m-RCA includes mid RCA; d-RCA includes distal RCA, PDA-RCA, PLB-RCA, s-RCA includes side branches of the RCA; p-LAD includes proximal LAD; m-LAD includes mid LAD; d-LAD includes distal LAD; s-LAD includes side branches of the LAD, Ramus intermedius, Diagonal 1, Diagonal 2; p-LCX includes proximal LCx; m-LCX includes mid LCx, d-LCX includes distal LCx, PDA-LCx and PLB-LCx; s-LCX includes side branches of LCx, Obtuse marginal 1 and Obtuse marginal 2**

Supplementary Table S3: Confusion matrix of the test set for the assignment of calcifications (number of calcifications) to the same vessel class. The model achieved excellent agreement with a Cohen's κ of 0.914 (95% CI 0.896-0.929) for assigning calcifications to the correct vessel.

	Multi-task DL model				
**Reference***	**RCA**	**LM**	**LAD**	**LCX**	**Total**
**RCA**	578	0	1	2	581
**LM**	0	79	25	6	110
**LAD**	1	31	746	7	785
**LCX**	8	19	5	304	336
**Total**	587	129	777	319	1812

*** RCA-right coronary artery, LM-left main, LAD-left anterior descending, LCX-left circumflex**

Supplementary Table S4: Performance of the model on the DISCHARGE test set for binary segmentation of coronary calcium and multiclass segmentation on the vessel level. Performance results are provided as interclass correlation coefficient (ICC), precision, sensitivity, specificity, and F1-score for CAC segmentation on volume (Vol.) and lesion level (No.).

**Binary CAC segmentation**	**Number of voxels/lesions**	**ICC (Vol./No.)**	**Precision (Vol./No.)**	**Sensitivity (Vol./No.)**	**Specificity (Vol./No.)**	**F1-score (Vol./No.)**
**CAC / No CAC**	191686/1976	0.93/0.94	0.93/0.79	0.89/0.89		0.91/0.84
**Binary CAC segmentation on vessel level**	**Number of voxels/lesions**	**ICC (Vol./No.)**	**Precision (Vol./No.)**	**Sensitivity (Vol./No.)**	**Specificity (Vol./No.)**	**F1-Score (Vol./No.)**
**RCA**	70455/674	0.81/0.92	0.99/0.98	1.0/0.99	0.99/0.99	0.99/0.99
**LM**	13140/133	0.67/0.7	0.64/0.61	0.67/0.72	0.97/0.97	0.66/0.66
**LAD**	83541/839	0.95/0.95	0.95/0.96	0.96/0.95	0.96/0.97	0.95/0.96
**LCX**	30462/387	0.83/0.89	0.94/0.95	0.89/0.9	0.99/0.99	0.92/0.93
**Multiclass CAC segmentation on vessel level**	**Number of voxel/lesions**	**Micro ICC* (Vol./No.)**	**Micro Precision* (Vol./No.)**	**Micro Sensitivity* (Vol./No.)**	**Micro Specificity* (Vol./No.)**	**Micro F1-Score* (Vol./No.)**
**Micro Vessel Score**	-	0.99/0.99	0.94/0.94	0.94/0.94	1.00/0.98	0.94/0.94

***Micro metrics are calculated from the assignment of correctly detected calcifications to the correct segment class (calcifications missed by the model are not included in the metric).**

Supplementary Table S5: Ablation experiment of the zero-CAC module. The ablation experiment evaluated the multi-task model without utilizing the proposed zero-CAC module. Confusion matrix shows the agreement of CVD risk estimates for the DISCHARGE test set with a Cohen's κ of 0.868 (95% CI: 0.831-0.898). Risk categorization was based on the Agatston score with risk category I: 0, II: [1,100), III: [100,300), IV: > 300.

Risk	I	II	III	IV	Total
**I**	141	50	2	1	194
**II**	1	113	6	1	121
**III**	0	0	63	3	66
**IV**	0	1	0	73	74
**Total**	142	164	71	78	455

Supplementary Table S6: Comparison of model performance with that of the second observer with first observer as reference standard on the DISCHARGE test set.
Performance results are provided as interclass correlation coefficient (ICC), precision, sensitivity, specificity and F1-score for CAC segmentation on the lesion level (No.) and volume level (Vol.).

SCCT Segment number	Adapted segment model	Number of calcified lesions	ICC (Vol.)		Precision (No.)		Sensitivity (No.)		Specificity (No.)		F1-Score (Vol.)		p-value†
			**Model**	**Obs. 2**	**Model**	**Obs. 2**	**Model**	**Obs. 2**	**Model**	**Obs. 2**	**Model**	**Obs. 2**	
**1**	**p-RCA**	346	0.88	0.92	0.88	0.95	0.94	0.79	0.97	0.99	0.81	0.82	0.003
**2**	**m-RCA**	131	0.35	0.88	0.68	0.55	0.58	0.69	0.98	0.96	0.5	0.65	0.034
**3, 4a, 4b**	**d-RCA**	182	0.69	0.96	0.86	0.82	0.92	0.89	0.99	0.98	0.73	0.83	0.567
**RCA-side**	**s-RCA**	15	0.0	0.73	-	0.73	0.00	0.73	1.00	1.00	0.0	0.68	0.066
**5**	**LM**	133	0.67	0.75	0.61	0.65	0.72	0.68	0.97	0.98	0.53	0.59	0.324
**6**	**p-LAD**	303	0.80	0.93	0.64	0.73	0.71	0.78	0.92	0.95	0.61	0.79	0.095
**7**	**m-LAD**	241	0.72	0.8	0.53	0.65	0.53	0.61	0.93	0.95	0.5	0.64	<0.001
**8**	**d-LAD**	134	0.48	0.8	0.71	0.66	0.50	0.64	0.99	0.98	0.52	0.61	0.081
**9, 10, 17**	**s-LAD**	160	0.85	0.95	0.76	0.89	0.72	0.82	0.98	0.99	0.64	0.84	<0.001
**11**	**p-LCX**	241	0.73	0.91	0.88	0.8	0.80	0.87	0.98	0.97	0.76	0.79	0.403
**13**	**m-LCX**	29	0.83	0.81	0.53	0.37	0.61	0.8	0.99	0.98	0.50	0.51	0.505
**15, 16a, 16b**	**d-LCX**	29	0.47	0.53	0.88	0.39	0.70	0.83	1.00	0.98	0.60	0.49	1.000
**12, 14**	**s-LCX**	89	0.67	0.42	0.71	0.96	0.71	0.29	0.99	1.00	0.54	0.34	0.002
**Binary CAC segmentation**	-	2033	0.91	1.00	0.86	0.79	0.88	0.95	1.00	1.00	0.87	0.96	0.006
			**Micro ICC* (Vol.)**		**Micro Precision* (No.)**		**Micro Sensitivity* (No.)**		**Micro Specificity* (No.)**		**Micro F1-Score* (No.)**		
**Micro segment score**	-	-	0.87	0.90	0.73	0.74	0.73	0.74	0.98	0.98	0.72	0.76	0.117

***Micro metrics are calculated based on the classification of correctly detected calcification into the correct segment class (missed calcification of the model are not included in the metric)**
†p-values estimated from F1-score (micro-F1-score) using Wilcoxon signed-rank test.

Supplementary Table S7: Performance of the standard U-Net model on the DISCHARGE test set for binary segmentation of coronary calcifications and multiclass segmentation on the segment level. Performance results are provided as interclass correlation coefficient (ICC), precision, sensitivity, specificity and F1-score for CAC segmentation on the volume (Vol.) and lesion level (No.).

SCCT Segment number	Adapted segment model	Number of calcified voxel/lesions	ICC (Vol./No.)	Precision (Vol./No.)	Sensitivity (Vol./No.)	Specificity (Vol./No.)	F1-Score (Vol./No.)
**1**	**p-RCA**	36886/346	0.73/0.58	0.66/0.71	1.00/0.99	0.88/0.91	0.65/0.58
**2**	**m-RCA**	16346/131	0.00/0.00	-/-	0.00/0.00	1.00/1.00	0.00/0.00
**3, 4a/4b**	**d-RCA**	16620/182	0.51/0.50	0.89/0.87	0.50/0.51	1.00/0.99	0.50/0.46
**RCA-side**	**s-RCA**	603/15	0.00/0.00	-/-	0.00/0.00	1.00/1.00	0.00/0.00
**5**	**LM**	13140/133	0.59/0.37	0.58/0.56	0.75/0.77	0.96/0.96	0.46/0.39
**6**	**p-LAD**	41787/303	0.77/0.49	0.65/0.59	0.78/0.70	0.87/0.91	0.67/0.53
**7**	**m-LAD**	24019/241	0.62/0.40	0.45/0.40	0.54/0.67	0.90/0.85	0.45/0.38
**8**	**d-LAD**	7903/134	0.03/0.05	-/-	0.00/0.00	1.00/1.00	0.00/0.00
**9, 10, 17**	**s-LAD**	9832/160	0.00/0.09	0.87/1.00	0.01/0.04	1.00/1.00	0.03/0.06
**11**	**p-LCX**	21363/241	0.63/0.29	0.61/0.54	0.85/0.88	0.93/0.89	0.53/0.41
**13**	**m-LCX**	3002/29	0.00/0.01	-/-	0.00/0.00	1.00/1.00	0.00/0.00
**15, 16a/16b**	**d-LCX**	1355/29	0.01/0.01	-/-	0.00/0.00	1.00/1.00	0.00/0.00
**12, 14**	**s-LCX**	4742/89	0.01/0.02	-/-	0.00/0.00	1.00/1.00	0.00/0.00
**Binary CAC segmentation**	-	197598/2033	0.87/0.80	0.79/0.60	0.88/0.89	1.00/1.00	0.83/0.72
		**Number of calcified voxel/lesions**	**Micro ICC* (Vol./No.)**	**Micro Precision* (Vol./No.)**	**Micro Sensitivity* (Vol./No.)**	**Micro Specificity* (Vol./No.)**	**Micro F1-Score* (Vol./No.)**
**Micro segment score**	-		0.76/0.65	0.62/0.58	0.62/0.58	0.97/0.97	0.62/0.58

***Micro metrics are calculated based on the classification of correctly detected calcification into the correct segment class (missed calcification of the model are not included in the metric)**

References

1. Kendall A, Badrinarayanan V, Cipolla R. Bayesian SegNet: Model Uncertainty in Deep Convolutional Encoder-Decoder Architectures for Scene Understanding. 2017.
2. Zegers P. Fisher Information Properties. Entropy. 2015 doi: 10.3390/e17074918.
3. Kirsch A, Gal Y. Unifying Approaches in Active Learning and Active Sampling via Fisher Information and Information-Theoretic Quantities. Trans Mach Learn Res. 2022;2022.
4. Fedorov A, Beichel R, Kalpathy-Cramer J, Finet J, Fillion-Robin JC, Pujol S, et al. 3D Slicer as an image computing platform for the Quantitative Imaging Network. Magn Reson Imaging. 2012;30(9):1323-41. doi: 10.1016/j.mri.2012.05.001.
5. Cipolla R, Gal Y, Kendall A. Multi-task Learning Using Uncertainty to Weigh Losses for Scene Geometry and Semantics. 2018 IEEE/CVF Conference on Computer Vision and Pattern Recognition (CVPR)2018. p. 7482-91.
6. Cohen J. Weighted kappa: nominal scale agreement with provision for scaled disagreement or partial credit. Psychol Bull. 1968;70(4):213-20. doi: 10.1037/h0026256.
